# Differential uptake of three clinically relevant allergens by human plasmacytoid dendritic cells

**DOI:** 10.1186/s12948-021-00163-8

**Published:** 2021-11-17

**Authors:** Noelle Zurmühl, Anna Schmitt, Ulrike Formentini, Johannes Weiss, Heike Appel, Klaus-Michael Debatin, Dorit Fabricius

**Affiliations:** 1 Department of Pediatrics, University Medical Center Ulm, Eythstr. 24, 89075 Ulm, Germany; 2 Department of Dermatology and Allergic Diseases, University Medical Center Ulm, Ulm, Germany; 3 Department of Otolaryngology, Ulm University, Ulm, Germany

**Keywords:** Plasmacytoid, Dendritic cell, Allergen uptake

## Abstract

**Background:**

Human plasmacytoid dendritic cells (pDC) have a dual role as interferon-producing and antigen-presenting cells. Their relevance for allergic diseases is controversial. and the impact of pDC on allergic immune responses is poorly understood.

**Methods:**

This in vitro study on human pDC isolated from peripheral blood was designed to compare side by side the uptake of three clinically relevant representative allergens: fluorochrome-labeled house dust mite Der p 1, Bee venom extract from Apis mellifera (Api) and the food allergen OVA analyzed flow cytometry and confocal microscopy.

**Results:**

We found that the internalization and its regulation by TLR9 ligation was significantly different between allergens in terms of time course and strength of uptake. Api and OVA uptake in pDC of healthy subjects was faster and reached higher levels than Der p 1 uptake. CpG ODN 2006 suppressed OVA uptake and to a lesser extent Der p 1, while Api internalization was not affected. All allergens colocalized with LAMP1 and EEA1, with Api being internalized particularly fast and reaching highest intracellular levels in pDC. Of note, we could not determine any specific differences in antigen uptake in allergic compared with healthy subjects.

**Conclusions:**

To our knowledge this is the first study that directly compares uptake regulation of clinically relevant inhalative, injective and food allergens in pDC. Our findings may help to explain differences in the onset and severity of allergic reactions as well as in the efficiency of AIT.

**Supplementary Information:**

The online version contains supplementary material available at 10.1186/s12948-021-00163-8.

## Key points

Side by side comparison of inhalative, injective and food allergen uptake by pDC

Uptake of allergens Der p 1, ovalbumin, bee venom significantly differs in pDC

Allergens colocalize with LAMP1 and EEA1 the fastest with bee venom.

## Introduction

Plasmacytoid dendritic cells (pDC) are a unique naturally occurring dendritic cell (DC) subset with two outstanding capabilities, one as interferon (IFN)-proåducing and the other as antigen-presenting cells (APC), thereby mediating both innate and adaptive immune responses [1, 2]. Apart from their particular role in mediating and promoting antiviral immune responses, they modulate immunity in autoimmune and neoplastic [3–5], but also in allergic diseases. Whether pDC play a rather tolerogenic [6, 7], or allergy-promoting role [8, 9] is controversial. Moreover, it is hypothesized that a primary defect of pDC function contributes to the development of asthma [10]. The latter may match observations of an inverse correlation of pDC blood counts with childhood respiratory tract infections and wheezing [11].

Although accounting for only 0.1–0.5% of peripheral blood cells, pDC produce more than 95% of type I IFN among circulating lymphocytes [12], of which IFN-alpha is the main representative. Allergic diseases have been shown to impair IFN-alpha production, contributing to the susceptibility of allergic patients to viral infections, particularly subjects with asthma [13, 14]. Interestingly, CpG oligodeoxynucleotides, which act via TLR9 as one of the TLRs mainly expressed in pDC, can modulate these proallergic effects and convert pDC-driven immune responses towards a Th1 phenotype [9].

The allergens ovalbumin (OVA, equals the allergen component Gal d 2) from hen’s egg, bee venom from Apis mellifera (Api) and the major allergen of house dust mite (HDM), Dermatophagoides pteronyssimus 1 (Der p 1), represent examples of the three major groups of allergens: food allergens, injective allergens and inhalative allergens, respectively. To what extent pDC internalize and process these allergens has not been elucidated yet. As for other antigens, it can be assumed that allergens are taken up and presented to other immune cells such as B and T cells. It has been described that antigen endocytosis by pDC in general is not as efficient as by conventional DC [15], but sufficiently effective to rapidly cross-present antigens, e.g. of viral origin, on MHC class I [16, 17].

IL-3, a cytokine mainly released by mast cells and activated T cells, is a key survival factor for pDC, and essential to keep this DC type in culture for up to 6 days [18]. IL-3 is the major cytokine promoting IL-4 and IL-13 expression and the most potent priming cytokine of basophils [19]. IL-4, and IL-13 are major mediators of allergy and asthma. They are produced by basophils and are released in large quantities after stimulation with IL-3 [19]. Therefore, these cytokines may affect pDC function in allergic settings.

For certain DC subsets such as monocyte-derived dendritic cells (moDC) allergen uptake has been described in great detail [20, 21], however respective data in pDC is lacking so far. A recent study on moDC revealed trafficking of the grass pollen allergen Phl p 5 and the sunflower allergen SF-nsLTP via early and late endosomal markers, Early Endosome Antigen 1 (EEA-1) and Lysosome-Associated Membrane protein 1 (LAMP-1) [21].

In the current study, we investigated the uptake of the three clinically relevant allergens Der p 1, purified Api and OVA by pDC. Using FACS analysis and confocal microscopy, we characterized the time course of their uptake and their colocalization with endosomal markers.

## Materials and methods

### Reagents and labeling of allergens

The TLR9 agonist CpG oligodeoxynucleotide ODN 2006 (CpG B) with the specific sequence 5’-TCG TCG TTT TGT CGT TTT GTC GTT-3’ and the cytokines IL-3, IL-4 and IL-13 were purchased from Miltenyi Biotec (Bergisch Gladbach, Germany). Unlabeled purified OVA was purchased from Thermo Fisher Scientific (Eugene, USA) and used in a subset of experiments after labeling (see below). Purified unlabeled house dust mite major allergen Der p 1 and unlabeled Apis mellifera bee venom extract were purchased from Citeq biologics (Groningen, the Netherlands). For analysis of allergen uptake, unlabeled allergens were stained with an Alexa Fluor 555 labeling kit, Cat. No. A30007 from Thermo Fisher Scientific. To enable proper labeling of Der p 1 with Alexa Fluor 555 Der p 1 had to be desalted, using Zeba™ Spin Desalting method (Thermo Fisher). Api was sufficiently labeled without desalting. Labeling of allergens was confirmed using a microplate reader Tecan Infinite PRO 200 (280 nm excitation, 555 nm emission). In addition, DQ™ ovalbumin, a self-quenched OVA conjugate labeled with BODIPY® FL dye (Molecular probes, provided by Thermo Fisher) was used to detect processed antigen. It exhibits bright green fluorescence upon proteolytic degradation. In addition, the Alexa Fluor 647 Ovalbumin conjugate (Molecular probes, provided by Thermo Fisher) was used to detect the amount of internalized OVA.

### Human blood samples, cell isolation and cell culture

The present study was approved by the Ethics Committee at Ulm University. Peripheral blood from healthy volunteers was acquired after obtaining informed consent. Mononuclear cells from peripheral blood (PBMC) were either isolated from fresh peripheral blood or buffy coats by Ficoll density gradient centrifugation or enriched via leukapheresis at the Institute of Transfusion Medicine, Ulm University. For the latter, 10% Acid-Citrate-Dextrose formulation A (ACD-A) was added as anticoagulant and cell viability optimized by adding 50% human plasma. Red blood cells were lysed using ACK lysis buffer (0.15 M NH_4_Cl, 10 mM KHCO_3_, 0.1 mM Na_2_EDTA, pH = 7.3). PDC were isolated by positive magnetic bead selection using the BDCA-4^+^ cell isolation kit (Miltenyi Biotec). Purity was usually > 90%, with pDC defined as lin-1^−^, BDCA-2^+^, CD123^++^. In some experiments, peripheral blood from individuals who were either allergic to HDM or Api where only limited blood volume was available, a panDC enrichment kit (Miltenyi Biotec) was used and pDC gated as above, with a fraction of enriched pDC of about 20–50%. For allergic subjects, age and sex matched volunteers served as controls. After isolation, pDC or panDC were cultured in AIM-V medium (Gibco, Thermo Fisher Scientific) in the presence of IL-3 (10 ng/ml for purified pDC, 20 ng/ml for panDC). 1 × 10^6^ cells/ml, 200 µl/well, were cultured in 96-well round-well plates for 16 h or 24 h at 37 ºC and 5% CO_2_ in the presence or absence of CpG ODN 2006 (2.5 µg/ml), IL-4 (100 ng/ml, equals 500 U/ml) or IL-13 (250 ng/ml, equals 100 U/ml) or allergens. Allergen concentrations were used as indicated and ranged between 10 and 50 µg/ml.

### Flow cytometry

V450, FITC, PE, PerCP-Cy5.5, APC, APC-H7 or PE-Cy7 labeled antibodies to CD45, lin-1, CD123, CD40, CD80, CD83, CD86, MHC class I and MHC class II were purchased from Becton Dickinson (Heidelberg, Germany), PE- or VioBlue labeled antibodies to BDCA-2 (CD303) from Miltenyi Biotec. Anti-CD45 APC was purchased from R&D Systems, Minneapolis, Minnesota, USA. Cells were harvested at the indicated time points and stained as previously described [22, 23]. Briefly, washed and resuspended pDC were surface-stained with previously determined antibody volumes, incubated for 14 min at room temperature in the dark, washed, spun down and resuspended for analysis. Identification of pDC was carried out by gating on lin-1^−^, CD123^++^ cells after culture since BDCA-2 is often internalized upon pDC activation [24]. For pDC gating strategy see Additional file 1: Fig. S1. Flow cytometric analysis was performed on an LSRII (Becton Dickinson Immunocytometry Systems, San Jose, CA). Alexa Fluor 555-labeled allergens were measured in the PE channel, Alexa Fluor 647-labeled OVA in the APC channel, DQ OVA BODIPY in the FITC channel. Data were analyzed using FlowJo software (version 10.5.3; Tree Star, Stanford, CA).

### Confocal microscopy

Alexa Fluor 488-labeled anti-LAMP-1 and an appropriate isotype control were purchased from Invitrogen (Carlsbad, CA, USA), Anti-EEA-1 Alexa Fluor 488 was derived from R&D Systems (Minneapolis, Minnesota, USA) and from Abcam (Cambridge, UK). Isolated pDC from buffy coats (BDCA-4 positive selection, Miltenyi Biotec. Purity > 90%) were cultured in AIM-V serum free medium in the presence of IL-3 (10 ng/ml), in 96-well round-well plates (200 µl, 5 × 10^5^ cells per well) with 40 µg/ml Alexa Fluor 555- labeled allergen Api extract, Der p 1 or OVA at 37 °C, 5% CO_2_, for either 90 min or 16 h. After transfer to 5 ml tubes and washing with 2 ml PBS, cells were centrifuged at 400 × g for 5 min and supernatants aspirated to a remaining volume of 200 µl. Cells were stained with 25 µl anti-CD123 APC (Miltenyi Biotec) and incubated for 15 min at room temperature in the dark. After washing and removal of supernatants, pDC were resuspended in 200 µl BSA 2% (Acros Organics, distributed by ThermoFisher Scientific) and incubated for 30 min on ice in the dark. After centrifugation at 400 g for 5 min, supernatants were completely aspirated and 100 µl fixation buffer (BD biosciences, Franklin Lakes, New Jersey, USA) was added. This was followed by incubation for 20 min at 4 °C. Cells were washed with 1 ml permeabilization/wash buffer, supernatants completely aspirated and cells taken up in 100 µl permeabilization/wash buffer (BD biosciences). For intracellular staining, 25 µl of either anti-EEA-1 Alexa Fluor 488 or Alexa Fluor 488-labeled anti-LAMP-1 were added, followed by incubation for 15 min at 4 °C in the dark. After washing, supernatants were aspirated, pDC resuspended in 150 µl PBS and then placed onto dried, Poly-D-Lysine- coated coverslips. After incubation for 90–120 min at 37 °C, PBS was aspirated from the coverslips and coverslips were dried at room temperature. DAPI for cell nucleus staining (Biolegend, San Diego, CA, USA) was added to the mounting medium (Vectashield, 1 ng/ml in PBS, Vector Laboratories, Burlingame, CA, USA) at a concentration of 1.5 µl DAPI in 1 ml. 5 µl of mounting medium were then placed on the slide and dried coverslips put onto the mounting medium (cells facing down). Coverslips were fixed using clear nail polish and let dry. For confocal microscopy, a Leica TCS SP8 inverse confocal microscope with an HC PL APO CS2 objective 63 × magnification was used. Images were collected and stored using Leica Application X (version 3.1.5.16308) software and processed using FIJI 2.1.0.

### Statistics

Summarized data are expressed either as box plots, with box central horizontal lines indicating medians, box borders representing interquartile ranges (IQR), and whiskers indicating minima and maxima. To determine statistical differences between nonparametric related samples, the Friedman test, for samples with unequal numbers, the Kruskal–Wallis-Test or Mixed-effects analysis was used, each followed by Dunn’s multiple comparisons test. To compare multiple related data sets, the 2way ANOVA, followed by Tukey’s multiple comparisons test were performed. For unrelated samples, Šídák multiple comparisons test was used. Results were considered significant with *p-*values < 0.05, and highly significant with *p-*values < 0.01.

## Results

### Allergen uptake in pDC is differentially regulated

**Fig. 1:**
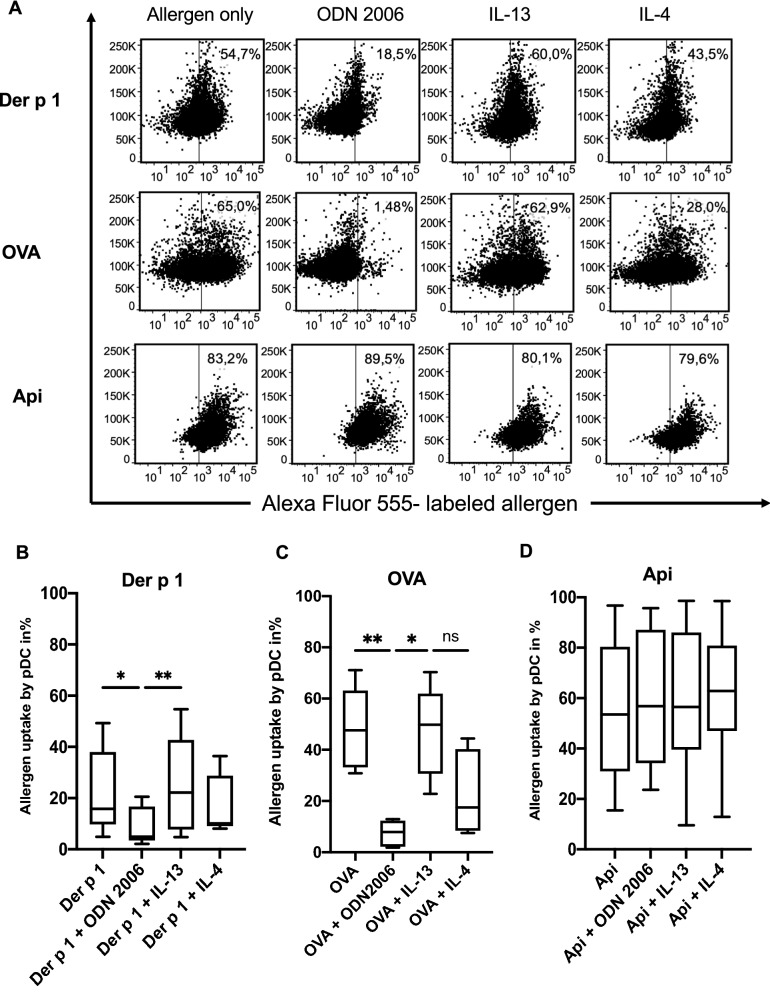
Regulation of allergen uptake of pDC varies among different allergens. PDC isolated from fresh peripheral blood or buffy coats by BDCA-4 positive magnetic bead selection were cultured overnight in AIM-V medium containing 10 ng/ml IL-3 in the presence or absence of CpG ODN 2006 (2,5 µg/ml), IL-4 (500 U/ml) or IL-13 (100 U/ml) or allergen as indicated. Prior to incubation, allergens were labeled using an Alexa Fluor 555 staining kit as described in Material and Methods. Allergen concentrations were 20 µg/ml for Der p 1 and 10 µg/ml for OVA and Api. After incubation, surface staining was performed with FITC conjugated Lin-1 and PerCP Cy5.5 conjugated CD123 and allergen uptake was analyzed by flow cytometry. PDC were defined as Lin-1^−^/CD123^++^ cells with pDC comprising 24–50%. **A** Dot plots show examples of allergen uptake regulation, which are summarized in (**B**–**D**), where box plots show percentages of allergen-positive pDC, central horizontal lines indicate medians, box borders represent IQR, whiskers indicate minima and maxima. **B** n = 6, **C** n = 5, **D** n = 8 experiments. Significance levels were assessed by Friedman test with p-values: ** p < 0.005 and * p < 0.05

The ability of pDC to take up small molecules was described before, but no specific focus was set on the quantitative uptake of clinically relevant allergens such as Api, Der p 1 and OVA. To assess allergen uptake into pDC of healthy individuals, pDC were isolated, cultured for 16 h with fluorochrome-labeled allergens in the presence or absence of TLR agonists or known pro-allergic cytokines, and analyzed via flow cytometry. Additional file 1: Fig. S1 shows the gating strategy. Allergen uptake during different culture settings varied depending on the allergens used (Fig. 1). Relevant internalization of Der p was detected in up to 55% of pDC, whereas OVA was internalized up to 65% and Api were taken by up to 80% of pDC (Fig. 1). With TLR9 agonist ODN 2006, a proven activator of pDC, the uptake of Der p 1 was markedly decreased. The interleukins IL-4 and IL-13, which imitate a pro-allergic cytokine milieu, did not affect the percentage of Der p 1 internalization. Similar to Der p1, OVA uptake was significantly suppressed in the presence of TLR9 agonist ODN 2006. OVA uptake is therefore strongly influenced by the activation of TLRs and the resulting downstream signals. Furthermore, OVA was by trend decreased in the presence of IL-4, which was not significant for OVA Alexa Fluor 555, but for commercially labeled OVA, DQ™ ovalbumin conjugated to BODIPY® FL dye, to detect processed antigen and Alexa Fluor 647-Ovalbumin conjugate, where ODN 2006 and IL-4 had strong inhibitory effects (Additional file 1: Fig. S2). In contrast, IL-13 had no impact on allergen uptake (Fig. 1C and Additional file 1: Fig. S2). Api uptake (Fig. 1D) was neither altered by the Th2-cytokines IL-4 and IL-13 nor by ODN 2006.

### Allergen uptake by pDC is an active process and has minor effects on pDC phenotype

**Fig. 2:**
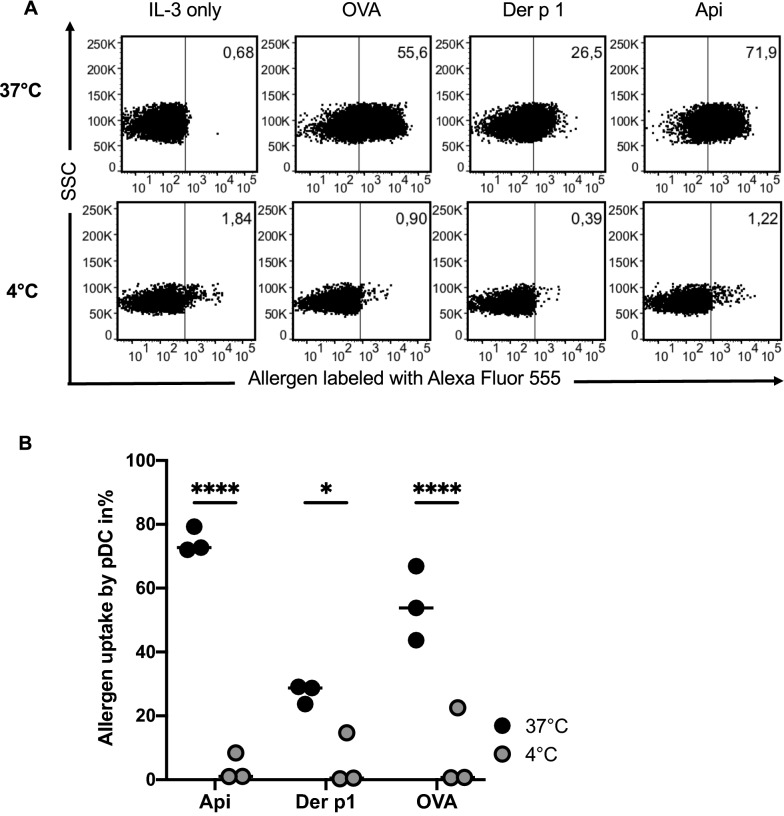
Allergen uptake into pDC is an active process. PDC were isolated from buffy coats by BDCA-4 positive selection. Lin-1^−^/BDCA-2^+^ staining revealed a pDC purity of > 95%. After overnight incubation at 4 °C or 37 °C in AIM-V medium containing 10 ng/ml IL-3 and 10 µg/ml Alexa Fluor 555 labeled allergens (Api, OVA or Der p 1) allergen uptake was analyzed by flow cytometry. **A** FACS dot plots show results from one representative experiment. **B** Individual results from three independent experiments show percentages of allergen positive pDC. Significance levels were assessed by Šídák's multiple comparisons test, matching each allergen across the two temperatures, with p-values: **** p < 0.00005 and * p < 0.05

To confirm that allergen uptake into pDC of healthy individuals takes place is an active process, pDC were isolated and cultured for 16 h either at 37 °C, or at 4 °C with allergens previously labeled with Alexa Fluor 555 as described. FACS analysis revealed significant intracellular antigen uptake by pDC only at 37 °C, but not at 4 °C, confirming the allergens Api, Der p 1 and OVA were actively taken up into pDC (Fig. 2). Moreover, we measured expression of surface markers CD40, CD80, CD83, CD86, MHC class I and MHC class II in the presence of allergens and pro-allergic cytokines IL-4 and IL-13 after 24 h, the time point with maximum allergen uptake. In the presence of IL-4, we observed a decrease of CD40, CD83 and MHC class I expression, with no significant change on CD80 or CD86 expression. Allergen uptake had only minor effects on pDC phenotype, only Api significantly enhanced CD80, Api plus IL- 4 enhanced expression of CD86, whereas Der p 1 plus IL-4 decreased CD80 expression. In contrast, IL-4-induced MHC class I suppression was even more pronounced after uptake of Api or Der p 1, a phenomenon, which was largely reversed by IL-13. Finally, expression of MHC class II was neither affected by allergens nor by proallergic cytokines after 24 h. (Additional file 1: Fig. S3).

### Allergen uptake by pDC occurs in a concentration- and time-dependent manner

**Fig. 3:**
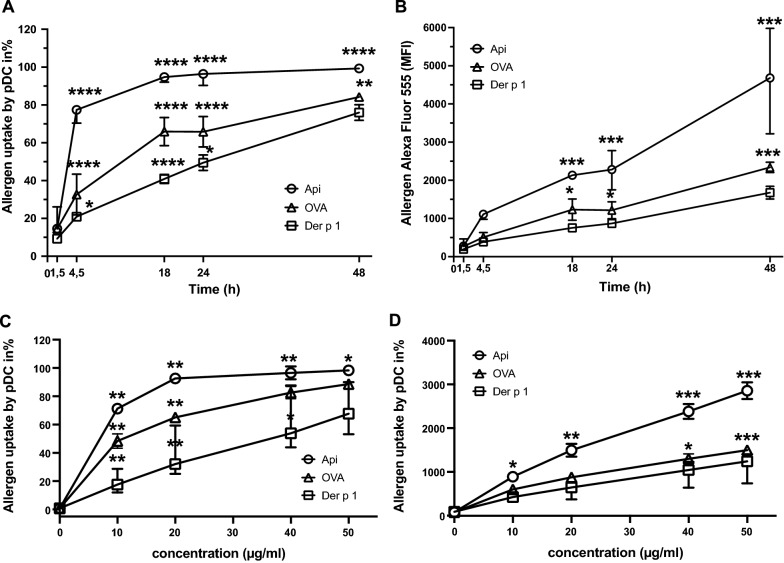
Time and concentration dependency of allergen uptake in pDC. PDC purified from buffy coats by BDCA4-positive selection were cultured in 96-round-well plates at a density of 1–1.5 × 10^5^ cells/well and cultured in AIM-V medium containing 10 ng/ml IL-3 and Alexa Fluor 555 labeled Api, Der p 1 or OVA. **A**, **B** PDC were incubated with 20 µg/ml allergen for 1.5 h, 4,5 h, 18 h, 24 h or 48 h. **C**, **D** Allergen concentrations were used as indicated and pDC cultured overnight. After incubation, surface staining was performed with FITC-conjugated Lin-1 and PerCP-Cy5.5-conjugated anti-CD123 as described and analyzed by flow cytometry. PDC were gated as Lin-1^−^ /CD123^++^. Allergen uptake was analyzed as percentage of Alexa Fluor 555-positive pDC and as median fluorescence intensity (MFI) in pDC. Allergen uptake by pDC from n = 3–7 (Der p 1) and n = 3 (Api and OVA) independent experiments is shown in **A**, **C** as median of percent, and in **B**, **D** as median of MFI. Error bars indicate ranges. (At some time points error bars are too small to be visible.) Significant differences were assessed using Tukeys multiple comparisons test with * p < 0.05, ** p < 0.005 and *** p < 0.0005

To compare the time course of uptake between the various allergens, a kinetics was performed and allergen internalization of Alexa Fluor 555-labeled allergens Api, Der p 1 and OVA determined at five different time points as indicated in Fig. 3. Notably, Api reached its uptake maximum earlier than OVA, whereas Der p 1 uptake occurred at the slowest rate. More than 95% of pDC took up Api within 24 h, faster reaching higher levels than with OVA and Der p 1 (Fig. 3A, ). This indicated that internalization by pDC varies among the three allergens tested. To investigate concentration dependency of uptake, Api, Der p 1 and OVA were used at different concentrations as indicated in Fig. 3C and . When comparing uptake within the same concentrations, Api reached higher uptake levels than OVA and Der p 1, with Der p 1 requiring the highest concentration to achieve similar uptake levels than the other allergens (Fig. 3C). This effect was somewhat more pronounced when looking at percentage of positive pDC than at the MFI (Fig. 3D).

**Fig. 4:**
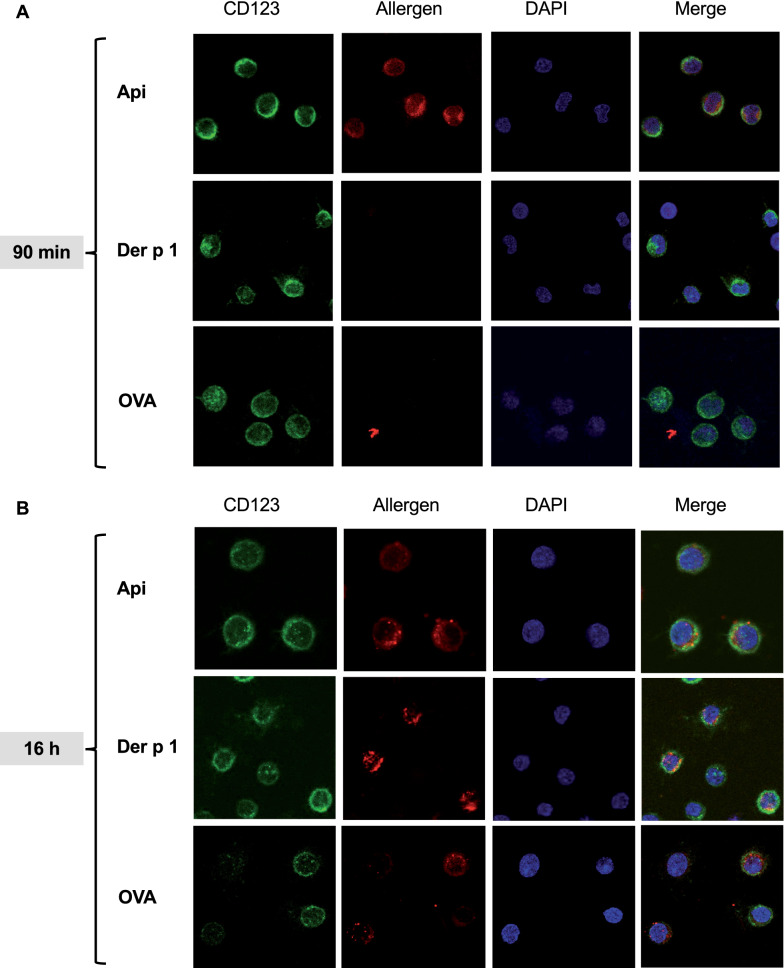
Api is internalized more rapidly by pDC than Der p 1 or OVA. PDC were isolated from buffy coats by positive selection. Then, they were cultured for either **A** 90 min or **B** 16 h in the presence of IL-3 at 10 ng/ml and 40 µg/ml Alexa Fluor 555-labeled Api, Der p 1 or OVA. After incubation, pDC were stained with CD123 APC and incubated for 15 min at room temperature in the dark. After washing with PBS, supernatants were completely aspirated and pDC resuspended in 200 µl BSA (2%) and incubated for 30 min on ice in the dark. After centrifugation, supernatants were completely aspirated and 100 µl fixation buffer was added. PDC were incubated for 20 min at 4 °C in the dark before washing with 1 ml permeabilization/wash buffer. Supernatants were removed and pDC resuspended in 100 µl permeabilization/wash buffer and incubated for 15 min at 4 °C in the dark. Cells were washed with 2 ml PBS, supernatants aspirated and pDC resuspended in 150 µl PBS. The cell suspension was then placed on dried, Poly-D-Lysine coated coverslips and incubated for 90–120 min at 37 °C. After incubation, PBS was aspirated from the coverslips and coverslips were let dry at room temperature. DAPI for cell nucleus staining was used as described and the dried coverslips were put onto the mounting medium cells facing down. Coverslips were fixed on the slide using clear nail polish and let dry. For confocal microscopy, a Leica TCS SP8 inverse confocal microscope was used, objective HC PL APO CS2 with 63 × magnification

To ensure that the data obtained by FACS indeed reflect internalization of allergens and to confirm that Api uptake occurred faster than uptake of Der p 1 or OVA, confocal microscopy was performed after allergens were incubated at different time points. We observed that after 90 min Api extract was taken up into pDC and could be clearly visualized intracellularly. In contrast, Der p 1 and OVA were not intracellularly detectable at this time point (Fig. 4). After 16 h, all three allergens could be visualized in the cytoplasm of pDC.

### Allergen in pDC colocalizes with endosomal and lysosomal markers

**Fig. 5:**
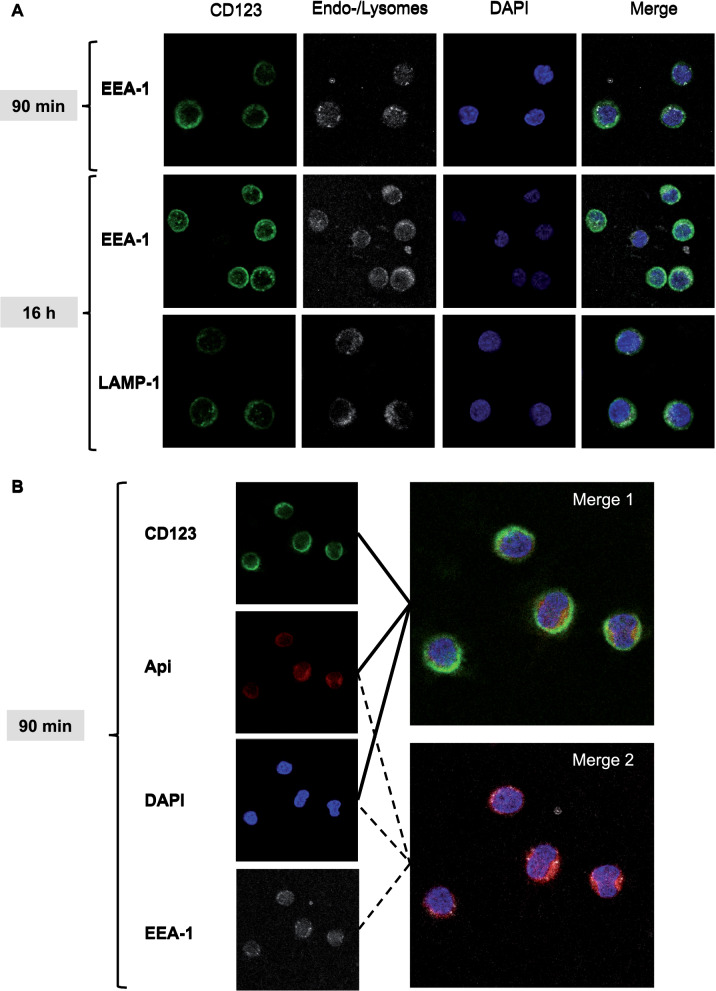
Bee venom extract Api can be colocalized with early endosome EEA1 after 90 min incubation. PDC were isolated from buffy coats by positive selection and then cultured for either 90 min or 16 h in the presence of 10 ng/ml IL-3 and 40 µg/ml Alexa Fluor 555-labeled bee venom, Der p 1 or OVA. After incubation, pDC were stained with anti-CD123 APC and incubated for 15 min at room temperature in the dark. After washing with PBS, supernatants were completely aspirated, pDC resuspended in 200 µl BSA (2%) and incubated for 30 min on ice in the dark. After centrifugation at 400 × g for 5 min, supernatants were completely aspirated and 100 µl fixation buffer was added. PDC were incubated for 20 min at 4 °C in the dark before washing with 1 ml permeabilization/wash buffer. Supernatants were removed and pDC resuspended in 100 µl permeabilization/wash buffer. For intracellular staining, 25 µl of either anti-EEA-1 or -LAMP-1 conjugated with Alexa Fluor 488 were added, followed by incubation for 15 min at 4 °C in the dark. PDC were washed with 2 ml PBS (400 × g, 5 min), supernatants aspirated and pDC resuspended in 150 µl PBS. The cell suspension was then placed on dried, Poly-D-Lysine coated coverslips and incubated for 90–120 min at 37 °C. After incubation, PBS was aspirated from the coverslips and coverslips were let dry at room temperature. DAPI for cell nucleus staining was used as described and the dried coverslips were put onto the mounting medium cells facing down. Coverslips were fixed on the slide using clear nail polish and let dry. For confocal microscopy, a Leica TCS SP8 inverse confocal microscope was used, objective HC PL APO CS2 with 63 × magnification. **A** Staining for EEA-1 and LAMP-1, **B** Intracellular localization of bee venom and EEA-1 after 90 min

**Fig. 6:**
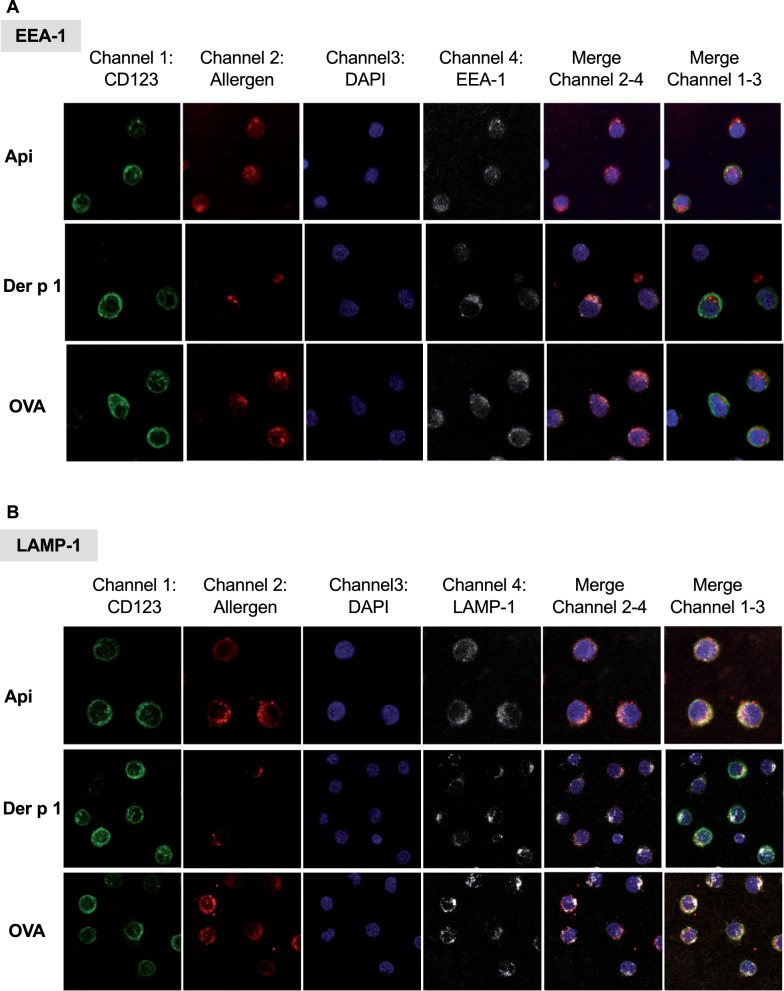
Bee venom, Der-p1 and OVA colocalize with EEA-1 and LAMP-1 after 16 h. PDC were isolated from buffy coats by positive selection and then cultured for 16 h in the presence of IL-3 at 10 ng/ml with 40 µg/ml Alexa Fluor 555-labeled bee venom, Der p 1 or OVA. After incubation, pDC were stained with anti-CD123 APC as described. After washing with PBS, supernatants were completely aspirated, pDC resuspended in 200 µl BSA (2%) and incubated for 30 min on ice in the dark. After centrifugation at 400 g for 5 min, supernatants were completely aspirated and 100 µl fixation buffer was added. PDC were incubated for 20 min at 4 °C in the dark before washing with 1 ml permeabilization/wash buffer. Supernatants were removed and pDC resuspended in 100 µl permeabilization/wash buffer. For intracellular staining, 25 µl of either anti-EEA-1 or -LAMP-1 conjugated with Alexa Fluor 488 were added followed by incubation for 15 min at 4 °C in the dark. After washing as described, pDC were placed on dried, Poly-D-Lysine coated coverslips and incubated for 90–120 min at 37 °C. Then PBS was aspirated from the coverslips and coverslips were let dry at room temperature. DAPI for cell nucleus staining was used as described and the dried coverslips were put onto the mounting medium. Coverslips were fixed on the slide using clear nail polish and let dry. For confocal microscopy, a Leica TCS SP8 inverse confocal microscope was used, objective HC PL APO CS2 with 63 × magnification. **A** Colocalization of EEA-1, **B** of LAMP-1 with indicated allergens

To characterize intracellular allergen transport in pDC, we investigated colocalization with early and late endosomal markers. Early Endosome Antigen 1 (EEA-1) and Lysosome-Associated Membrane Protein 1 (LAMP-1), the former associated with mildly acidic endosomes, the latter with more acidic endosomes and lysosomes [21] could both be visualized in pDC (Fig. 5). EEA-1 staining was already visible 90 min after culture, whereas LAMP-1 was visible after 16 h, with EEA-1 being also detectable at this time point (Fig. 5A). Since Api was internalized by pDC after 90 min (see above), this allergen could be detected in the intracellular compartment at the same time as EEA-1 became detectable (Fig. 5B). Der p 1 and OVA uptake could be visualized in pDC at 16 h, with Api still being visible at this time point (Fig. 6). While Api and OVA appeared to completely colocalize with EEA-1 after 16 h, Der p 1 showed a partial colocalization only with this endosomal marker (Fig. 6A). Similarly, complete colocalization with LAMP-1 was detectable for Api and OVA, but only partial colocalization for Der p 1 (Fig. 6B).

### No significant differences in allergen uptake between allergic and healthy individuals

**Fig. 7:**
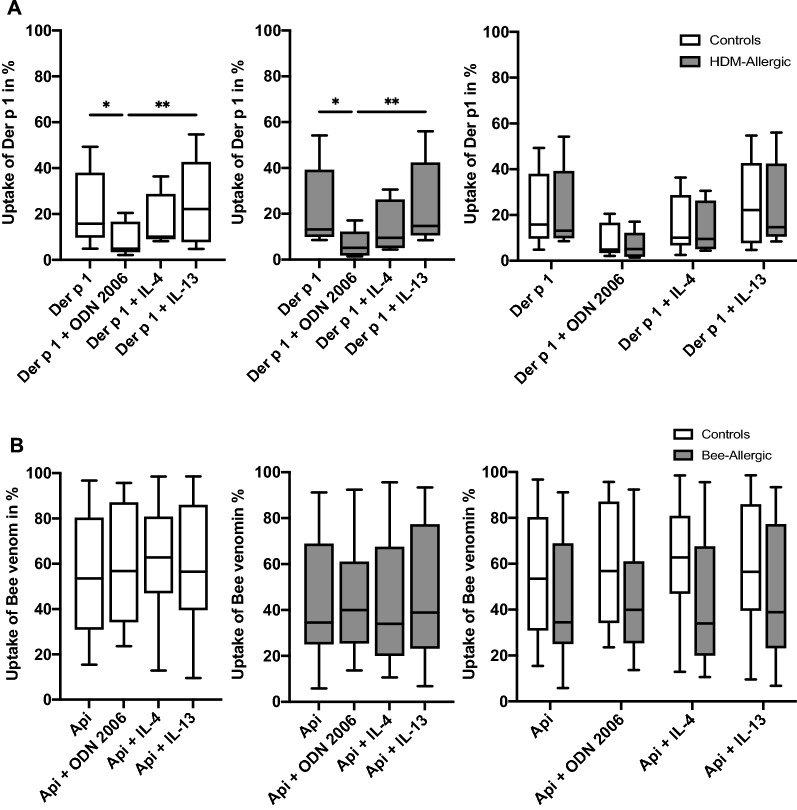
ODN 2006 suppresses uptake of Der p 1, but not Api in healthy and allergic subjects. PBMC from fresh blood samples were enriched for panDC and cultured overnight in AIMV medium containing 20 ng/ml IL-3 in the presence or absence of CpG ODN 2006 (2,5 µg/ml), IL-4 (500 U/ml) or IL-13 (100 U/ml) or allergen as indicated. Prior to incubation allergens were labeled using an Alexa Fluor 555 staining kit as described. Allergen concentrations were 20 µg/ml for Der p 1 and 10 µg/ml for bee venom. After incubation, allergen uptake was analyzed using flow cytometry. Frequencies of pDCs in the DC-enriched samples reached 24–50% as defined by Lin-1^−^/CD123^++^ staining. **A** Results from healthy controls without house dust mite (HDM) allergy (white boxes, n = 6) and individuals allergic to HDM (grey boxes, n = 5) are shown. **B** Data from healthy controls without bee allergy (white boxes, n = 8) and individuals allergic to bee venom (grey boxes, n = 12) are shown. Box plots illustrate percentages of allergen-positive pDC, central horizontal lines indicate medians, box borders represent IQR, whiskers indicate minima and maxima. Significance levels were **p < 0.005 and *p < 0.05, assessed by Friedman test, followed by Dunn’s multiple comparisons test

To determine potential differences between allergic and healthy individuals with regard to allergen uptake, pDC enriched from peripheral blood were incubated with the allergens Der p 1 or Api from allergic subjects and internalization of allergen was compared with that by pDC from healthy controls. FACS analysis was performed by gating on pDC as described. We observed a suppression of Der p 1 uptake by ODN 2006 both in pDC from healthy and allergic individuals, with no significant differences between both groups (Fig. 7A). Api, which was overall internalized by a higher percentage of pDC than Der p 1, both in healthy and allergic subjects, was not affected by the different stimulation patterns. Similarly, no significant differences were found between healthy control subjects and individuals allergic to Api, although here a trend towards a lower rate of antigen uptake in allergic individuals may be stated.

## Discussion

Several studies have been executed to characterize the role of DC in allergic diseases and tolerance induction in animal models or by investigating in vitro-generated monocyte-derived DC (moDC). Uptake of allergens such as OVA or Api has been investigated in mouse models and in murine bone marrow-derived pDC [25, 26] or moDC [27, 28]. However, detailed studies on human pDC that compare quantitative internalization of various allergens have been lacking so far.

PDC are capable of antigen presentation both to CD4 + and CD8 + T cells after they have been licensed to do so, e.g. via pattern recognition receptor activation [29, 30]. Whether this capacity contributes to an allergic inflammatory response or, to the contrary, may even dampen such a response, remains unclear to date. Recent literature points towards a tolerogenic role of pDC [6, 7] by regulating tolerance to self and nonself antigens [10], e.g. by TLR9 mediated regulatory T cell induction [31]. However, the contribution of pDC to either a pro- or an antiinflammatory response may not only depend on co-stimulation, but may also vary between different allergens.

In this work we performed a detailed side-by-side comparison of the uptake of three clinically relevant allergens by human pDC from peripheral blood, revealing differences in uptake quantities and time courses depending on the allergen subtype. Quantity and time course of allergen uptake showed differences between Der p 1, OVA and Api. Bee venom extract Api was internalized at the fastest rate and reached the highest intracellular levels compared with the other allergens tested. After as little as 90 min, Api was intracellularly detectable in pDC, when Der p 1 and OVA had not yet entered the intracellular compartment. It may be speculated that the magnitude of allergen uptake and kinetics affect the allergic immune response and tolerance induction, but this has been not addressed in recent publications to date. Also, the impact that pDC may have on allergies may rather depend on the way allergens are processed and presented, rather than on the quantity of their uptake.

For other DC such as moDC and Langerhans cells, a dose and time dependency of allergen uptake has been demonstrated [21, 32], with 10 µg/ml being the minimum of detectable allergen. In pDC, we observed a higher uptake of the allergens OVA and Api compared to Der p 1. Similarly, others observed a higher internalization of a food allergen versus an inhalative allergen using moDC incubated with sunflower LTP versus grass pollen allergen [21]. Moreover, for OVA we could demonstrate that it is proteolytically degraded in pDC after its uptake. Even though Der p 1 was the allergen with the lowest uptake in our study, Der p 1-pulsed pDC have been shown to dysregulate T cell responses derived from HDM-sensitized allergic patients, but not from heathy subjects, which may contribute to a steady state in healthy donors, but to allergenic Th2 responses in allergic donors [33]. For bee venom extract (Api) up to now 12 allergen components have been defined (http://www.allergen.org/viewallergen.php, accessed Oct 10th 2021). Api m 4, comprising about 50% of dry weight of bee extract [34] and the major allergen Api m1 are the smallest of the three allergens investigated here, with a molecular weight of 3 and 16 kDa, respectively, while Der p 1 has 24 kDa, the allergen Gal d 2, with the biochemical name OVA, has 44 kDa (http://www.allergen.org/viewallergen.php, accessed Oct 10th 2021). The small molecule size of Api components may therefore speed up internalization.

Another aspect which can influence allergen uptake are different extents of posttranslational modification such as allergen glycosylation which has been described for many allergens [35] including Api [36], OVA [37] and Der p 1. Glycosylation has been shown to affect Der p 1 internalization in moDC [20] but may also affect other allergens. Also pDC express mannose receptors [38]. To our knowledge, there is no publication on direct comparison of the effect of glycosylation patterns of Api, OVA and Der p 1, but it stands to reason that variable glycosylation patterns may result in different time course and quantities of allergen uptake.

It is yet unclear, which receptors mediate uptake of which allergen subtype into pDC. It is likely that some allergens use not only one but a variety of receptors for receptor-mediated endocytosis. For Der p 1 uptake in moDC, not only involvement of the mannose receptor [39], but also a role of ”Dendritic cell-specific **i**ntercellular adhesion molecule-3-grabbing non-integrin” (DC-SIGN) [40] has been described. For pDC no such studies have been carried out so far. In macrophages, Api m 1 (phospholipase A2) is recognized by mannose receptors [41]. Mannose receptors have been shown to mediate uptake of glycosylated antigens in myeloid DC subsets [42], which are involved in mediating Der p 1 uptake in DC subsets [43]. For pDC, this uptake mechanism for allergens has not been demonstrated yet.

In addition, simultaneous TLR9 stimulation in our study inhibited internalization and processing of OVA and Der p 1 uptake, indicating a relevant modulation by the TLR signaling pathway. It is well known that TLR7/9 signaling affects the overall impact of pDC on other immune cells, primarily T cells. It has been shown that TLR9 affects allergen presentation, and that TLR9 signaling preferably induces nonallergic Th1 cell differentiation [44]. For grass pollen it was described that pDC from patients with upper airway allergy induced allergen-dependent T-cell proliferation and Th2 cytokine production in the presence of grass pollen extract in coculture with CD4^+^ T cells [9]. In contrast, pDC activated with CpG ODN 2216 inhibited allergen-dependent proliferation of Th2 cells and markedly increased Th1 cytokine and IFN-gamma production [9]. As recently shown for tumor antigens [45], in the current study CpG ODN 2006 inhibited the uptake of the inhalative allergen Der p 1 and the food allergen OVA. These effects might point to a regulatory role of TLR9 agonists, an effect, which may be therapeutically relevant [46]. Since TLR9 ligands have been considered to be useful as therapeutic agents including as adjuvants in AIT formulations [47, 48], our results suggest they may not have the same effects in all types of allergy.

Of note, while internalization and processing of OVA and Der p 1 uptake was inhibited by TLR9 stimulation, Api uptake was not affected. The finding is surprising, since antigen uptake in general is reduced by soluble TLR ligands [49, 50] at least when it is mediated by the FcgammaRII receptor [51]. One reason why Api uptake was not affected by TLR9 may be due to the very quick internalization that took place before TLR9 ligation could be effective, since TLR9 ligands have to enter the endosomes to interact with TLR9. Due to the small size of the main bee venom components as described above, Api may utilize a wider range of uptake mechanisms than the other analyzed allergens.

Another reason why bee venom uptake follows a different kinetics than other allergens may be the unique binding properties of certain Api components. In an in vitro model with moDC, bee venom phospholipase A2 was shown to directly interact with cell membranes, which enabled internalization of a large quantity of antigen [52]. In addition, bee venom phospholipase A2 has binding properties through hydrophobic and electrostatic interaction with anionic phospholipids suggestive of a suitable tumor antigen vector [52, 53]. These outstanding properties may explain the lack of effect of TLR9 ligation.

Clearly, our descriptive data set cannot resolve these mechanistic possibilities but they do provide a basis for future mechanistic studies.

Regarding the impact of pro-allergic cytokines on allergen uptake by pDC, IL-4 decreased only the internalization of the allergen OVA and possibly Der p 1. In contrast, IL-4 did not affect uptake of Api. IL-4 and IL-13 are major mediators of allergy and asthma. They are produced by basophils and are released in large quantities after stimulation with IL-3 [19], a cytokine that pDC also need for survival [18]. One may therefore assume that uptake of allergens is basically controlled by these three cytokines, which thereby indirectly contribute to their allergenic effects.

To confirm that uptake of OVA, Der p 1 and Api is an active process we compared allergen internalization at 4° and 37 °C and observed intracellular allergen only after incubation at 37 °C. This is in line with observations in moDC, where grass pollen and sun flower allergens Phl p 5 and SF-ns-LTP used at similar concentrations were not internalized into moDC at 4 °C [21]. Instead, allergen particles were detected at the cell surface by confocal microscopy. In our study, we analyzed these temperature experiments by flow cytometry and saw no detectable labeling of pDC at 4 °C, which may be explained by a lower binding of allergen to the pDC surface.

With respect to pDC surface molecule expression, allergen uptake after 24 h had only minor effects on pDC phenotype Api by itself enhanced CD80 expression and Api plus IL- 4 increased expression of CD86, whereas Der p 1 plus IL-4 decreased CD80. In contrast, the presence of IL-4 significantly decreased CD40, CD83 and MHC class I expression, with MHC class I expression even more suppressed with additional allergens Der p 1 or Api. MHC class I suppression by allergens may hinder a crosstalk with CD8 + T cells and therefore indirectly favor a proallergic Th2 response. Expression of MHC class II, constitutively expressed on pDC at high levels, was neither affected by allergens, nor by IL-13 or IL-4. These findings support earlier observations that phenotypic changes in pDC are primarily TLR-mediated [23, 54] or occur after CD40 ligation [55]. HDM allergens have been described to have stimulatory TLR-mediated effects on DC subsets other than pDC, but these effects may have been induced at least in part by contaminating microbial compounds such as LPS which activates TLR4 [56]. Also, in the presence of CpG ODN, adding IL-4 or IL-13 enhances CD86, decreases MHC class I and increases MHC class II in pDC [57]. Other authors used a cytokine cocktail to stimulate pDC including IL-3, IL-4, anti-IL-12 and anti-IFN-gamma to imitate Th2-like conditions and compared its effect to a Th1 cocktail comprising IL-3, anti-IL-4, IL-12 and IFN-gamma. Among the obtained results was a reduction of MHC class II and costimulatory molecules such as CD86 as well as suppression of the proinflammatory cytokines IL-6, TNF-alpha and IFN-alpha by the Th2-like cytokine cocktail [58].

The observed differences in time course and quantity of allergen uptake in pDC may be clinically relevant. The typical allergic response to bee venom apart from isolated skin symptoms is anaphylaxis, which occurs with variable severity within minutes after the bee sting [59], and which is usually attributed to an IgE-mediated reaction that leads to release of mediators from mast cells and basophiles. However, since pDC are directly present at the site of the bee sting by circulating in peripheral blood and are capable of quick internalization of Api, they may contribute to the reaction. In contrast, after hen's egg consumption, allergen uptake including OVA may be mediated by a greater variety of APC including pDC in the gut or in the skin, particularly with defective skin barrier. This may result in a wide range of time courses and symptoms including pollen-related food syndromes, with anaphylaxis being one of many possibilities of reactions, and with a higher prevalence in children [60]. On the contrary, a reaction to house dust mite allergen Der p 1 does not occur in form of an anaphylactic reaction but rather in allergic rhinoconjunctivitis or asthma [61].

After prolonged incubation times up to 16 h all allergens colocalized with EEA-1 and LAMP-1 in human pDC. Since these are markers for early and late endosomes respectively, our results suggest that both endosomal compartments are involved in allergen processing. This is in line with studies in moDC, in which grass pollen, birch pollen and sunflower allergens also colocalized with EEA-1 and LAMP-1 [21, 62]. However, trafficking to EEA-1 was detected already after 15 min and to LAMP-1 after 30–45 min, which suggests allergen arrival at late endosomes occurs earlier in moDC than in pDC.

It is known that in individuals suffering from hymenoptera venom allergy, numeric and phenotypic changes in blood pDC are occurring during allergen-specific immunotherapy [63]. These findings are in line with the observation of a decrease of circulating pDC and phenotypic changes in patients with HDM allergy during subcutaneous immunotherapy [64]. Furthermore, it has been described that pDC are generally critical for tolerance induction by inducing Treg cells in a mouse model for uptake and presentation of oral allergen OVA [65].

Based on these differences, we hypothesized that similar differences may be present for allergen uptake by pDC. Nevertheless, in our hands, allergen uptake was not significantly different between pDC from healthy versus allergic subjects. This again is in line with observations by independent groups, who demonstrated that pDC from allergic and healthy individuals internalized Der p 1 without significant differences [33]. Similar results were described in moDC from individuals allergic to grass pollen allergen [21], or from individuals allergic to birch pollen [62], in all of which no major differences were found in comparison with healthy controls. Therefore, not quantity, but time course, processing and presentation of allergens in dendritic cells, activation status as well as the cytokine milieu and genetic factors may be more decisive for distinguishing allergic from healthy individuals.

In summary, our findings strengthen previous observations suggesting a more prominent role of pDC in allergy pathogenesis than generally anticipated. In our study we compared the uptake kinetics of representative inhalative, injective and food allergens by human pDC, namely Der p 1, Api extract and OVA. In general, internalization was significantly different between allergens in terms of time course and strength of uptake, as well as its regulation by TLR9 ligation. All allergens colocalized with LAMP1 and EEA1, Api was internalized particularly fast and reached high intracellular levels in pDC. Our findings therefore suggest peripherally circulating pDC play a major role for the uptake and processing of allergen. Although we could not determine specific differences in allergen uptake in allergic compared with healthy subjects, we observed variations between the various antigens themselves, which may explain disparities in the onset and severity of allergic reactions and may also be relevant for hymenoptera AIT, where bee or wasp venom is subcutaneously injected. Further studies are required to shed more light into the long-time underestimated role of pDC in allergic disease and tolerance induction.

## Supplementary Information

**Additional file 1: Figure S1**. Gating strategy of pDC analysis. PDC isolated from fresh peripheral blood, buffy coats or leukapheresis products by BDCA-4 positive magnetic bead selection were analyzed at day 0 for purity using flow cytometry. For this purpose, cells were stained with Lin-1 FITC, a lineage cocktail including antibodies to CD3, CD14, CD19, CD20, CD56) and with anti-BDCA-2 PE, with pDC being defined as Lin-1^neg^/BDCA-2pos cells. If purity was > 95% pDC were analyzed without further gating after culture. If purity was <95%, cultured pDC, which tend to downregulate BDCA-2, were stained with Lin-1 FITC and anti-CD123 PerCPCy5.5 and gated as Lin-1^neg^/ CD123^highly pos^ cells. **Figure S2**. CpG-ODN and IL-4 suppress OVA-uptake. PDC isolated from leukapheresis products by BDCA-4 positive magnetic bead selection with a purity of 95% were cultured in 10 ng/ml IL-3-containing AIM-V medium for 16h in the presence or absence of allergens, CpG ODN 2006 (2.5 μg/ml), IL-4 (500 U/ml) or IL-13 (100 U/ml). Allergens included DQ OVA Bodipy or Alexa Fluor 647-labeled OVA at a final concentration of 30 μg/ml. After incubation, OVA uptake (OVA Alexa 647) and OVA processing (DQ OVA Bodipy) was quantified using flow cytometry. (A) Dot plots show one out of at least 6 representative experiments. (B) Box plots show percentage of allergen-positive pDC, central horizontal lines indicate medians, box borders represent IQR, whiskers indicate minima and maxima, n = 5-12 experiments. Significance levels were **** p < 0.00005 and * p < 0.05, assessed with Kruskal-Wallis test, followed by Dunn‘s multiple comparisons test. **Figure S3**. Allergen exposure has only minor effects on pDC phenotype. Isolated pDC were incubated for 24 h in 10 ng/ml IL-3 containing AIM-V medium with Der p 1 (20 μg/ml) or Api (bee venom extract, 10 μg/ml), IL-4 (500 U/ml) or IL-13 (100 U/ml) or combinations as indicated and measured by flow cytometry. CD40, CD80, CD83, CD86 , MHC class I and MHC class II expression is shown as mean fluorescence intensity (MFI) relative to non-stimulated cells (medium), box plot central lines represent medians, box borders show IQR, whiskers indicate minima and maxima, dots represent individual values of at least 3 independent experiments. Significant differences between medium and various stimuli were assessed with Kruskal-Wallis test or Mixed effects analysis, followed by Dunett’s multiple comparisons test comparing all culture conditions to non-stimulated cells (medium), significance level was p < 0.05.

## Data Availability

The datasets used and/or analyzed during the current study are available from the corresponding author on reasonable request.

## Abbreviations

pDC: Plasmacytoid dendritic cells
Api: Apis mellifera (bee venom extract)
BDCA: Blood dendritic cell antigen
ODN: Oligodeoxyribonucleotide
Der p 1: Dermatophagoides pteronyssimus major allergen 1
MFI: Mean fluorecence intensity
IQR: Interquartile range
OVA: Ovalbumin

## References

[1] O'Keeffe M, Mok WH, Radford KJ (2015). Human dendritic cell subsets and function in health and disease. Cell Mol Life Sci.

[2] Swiecki M, Colonna M (2015). The multifaceted biology of plasmacytoid dendritic cells. Nat Rev Immunol.

[3] Reizis B, Bunin A, Ghosh HS, Lewis KL, Sisirak V (2011). Plasmacytoid dendritic cells: recent progress and open questions. Annu Rev Immunol.

[4] Gilliet M, Cao W, Liu YJ (2008). Plasmacytoid dendritic cells: sensing nucleic acids in viral infection and autoimmune diseases. Nat Rev Immunol.

[5] Schettini J, Mukherjee P (2008). Physiological role of plasmacytoid dendritic cells and their potential use in cancer immunity. Clin Dev Immunol.

[6] de Heer HJ, Hammad H, Soullie T, Hijdra D, Vos N, Willart MA, Hoogsteden HC, Lambrecht BN (2004). Essential role of lung plasmacytoid dendritic cells in preventing asthmatic reactions to harmless inhaled antigen. J Exp Med.

[7] Kool M, van Nimwegen M, Willart MA, Muskens F, Boon L, Smit JJ, Coyle A, Clausen BE, Hoogsteden HC, Lambrecht BN, Hammad H (2009). An anti-inflammatory role for plasmacytoid dendritic cells in allergic airway inflammation. J Immunol.

[8] Chairakaki AD, Saridaki MI, Pyrillou K, Mouratis MA, Koltsida O, Walton RP, Bartlett NW, Stavropoulos A, Boon L, Rovina N, Papadopoulos NG, Johnston SL, Andreakos E (2018). Plasmacytoid dendritic cells drive acute asthma exacerbations. J Allergy Clin Immunol.

[9] Farkas L, Kvale EO, Johansen FE, Jahnsen FL, Lund-Johansen F (2004). Plasmacytoid dendritic cells activate allergen-specific TH2 memory cells: modulation by CpG oligodeoxynucleotides. J Allergy Clin Immunol.

[10] Lynch JP, Mazzone SB, Rogers MJ, Arikkatt JJ, Loh Z, Pritchard AL, Upham JW, Phipps S (2014). The plasmacytoid dendritic cell: at the cross-roads in asthma. Eur Respir J.

[11] Upham JW, Zhang G, Rate A, Yerkovich ST, Kusel M, Sly PD, Holt PG (2009). Plasmacytoid dendritic cells during infancy are inversely associated with childhood respiratory tract infections and wheezing. J Allergy Clin Immunol.

[12] Siegal FP, Kadowaki N, Shodell M, Fitzgerald-Bocarsly PA, Shah K, Ho S, Antonenko S, Liu YJ (1999). The nature of the principal type 1 interferon-producing cells in human blood. Science.

[13] Gill MA, Bajwa G, George TA, Dong CC, Dougherty II, Jiang N, Gan VN, Gruchalla RS (2010). Counterregulation between the FcepsilonRI pathway and antiviral responses in human plasmacytoid dendritic cells. J Immunol.

[14] Tversky JR, Bieneman AP, Chichester KL, Hamilton RG, Schroeder JT (2010). Subcutaneous allergen immunotherapy restores human dendritic cell innate immune function. Clin Exp Allergy.

[15] Colonna M, Trinchieri G, Liu YJ (2004). Plasmacytoid dendritic cells in immunity. Nat Immunol.

[16] Di Pucchio T, Chatterjee B, Smed-Sorensen A, Clayton S, Palazzo A, Montes M, Xue Y, Mellman I, Banchereau J, Connolly JE (2008). Direct proteasome-independent cross-presentation of viral antigen by plasmacytoid dendritic cells on major histocompatibility complex class I. Nat Immunol.

[17] Hoeffel G, Ripoche AC, Matheoud D, Nascimbeni M, Escriou N, Lebon P, Heshmati F, Guillet JG, Gannage M, Caillat-Zucman S, Casartelli N, Schwartz O, De la Salle H, Hanau D, Hosmalin A, Maranon C (2007). Antigen crosspresentation by human plasmacytoid dendritic cells. Immunity.

[18] Grouard G, Rissoan MC, Filgueira L, Durand I, Banchereau J, Liu YJ (1997). The enigmatic plasmacytoid T cells develop into dendritic cells with interleukin (IL)-3 and CD40-ligand. J Exp Med.

[19] Tschopp CM, Spiegl N, Didichenko S, Lutmann W, Julius P, Virchow JC, Hack CE, Dahinden CA (2006). Granzyme B, a novel mediator of allergic inflammation: its induction and release in blood basophils and human asthma. Blood.

[20] Al-Ghouleh A, Johal R, Sharquie IK, Emara M, Harrington H, Shakib F, Ghaemmaghami AM (2012). The glycosylation pattern of common allergens: the recognition and uptake of Der p 1 by epithelial and dendritic cells is carbohydrate dependent. PLoS ONE.

[21] Ashjaei K, Palmberger D, Bublin M, Bajna E, Breiteneder H, Grabherr R, Ellinger I, Hoffmann-Sommergruber K (2015). Atopic donor status does not influence the uptake of the major grass pollen allergen, Phl p 5, by dendritic cells. J Immunol Methods.

[22] Fabricius D, Neubauer M, Mandel B, Schutz C, Viardot A, Vollmer A, Jahrsdorfer B, Debatin KM (2010). Prostaglandin E2 inhibits IFN-alpha secretion and Th1 costimulation by human plasmacytoid dendritic cells via E-prostanoid 2 and E-prostanoid 4 receptor engagement. J Immunol.

[23] Fabricius D, O'Dorisio M, Blackwell S, Jahrsdorfer B (2006). Human plasmacytoid dendritic cell function: inhibition of IFN-alpha secretion and modulation of immune phenotype by vasoactive intestinal peptide. J Immunol.

[24] Dzionek A, Sohma Y, Nagafune J, Cella M, Colonna M, Facchetti F, Gunther G, Johnston I, Lanzavecchia A, Nagasaka T, Okada T, Vermi W, Winkels G, Yamamoto T, Zysk M, Yamaguchi Y, Schmitz J (2001). BDCA-2, a novel plasmacytoid dendritic cell-specific type II C-type lectin, mediates antigen capture and is a potent inhibitor of interferon alpha/beta induction. J Exp Med.

[25] Kool M, Geurtsvankessel C, Muskens F, Madeira FB, van Nimwegen M, Kuipers H, Thielemans K, Hoogsteden HC, Hammad H, Lambrecht BN (2011). Facilitated antigen uptake and timed exposure to TLR ligands dictate the antigen-presenting potential of plasmacytoid DCs. J Leukoc Biol.

[26] Lee HS, Chung SH, Song MY, Kim SS, Shin HD, Shim WJ, Han AR, Lee JS (2008). Effects of bee venom on the maturation of murine dendritic cells stimulated by LPS. J Ethnopharmacol.

[27] Hilmenyuk T, Bellinghausen I, Heydenreich B, Ilchmann A, Toda M, Grabbe S, Saloga J (2010). Effects of glycation of the model food allergen ovalbumin on antigen uptake and presentation by human dendritic cells. Immunology.

[28] Zhang Y, Luo Y, Li W, Liu J, Chen M, Gu H, Wang B, Yao X (2016). DC-SIGN promotes allergen uptake and activation of dendritic cells in patients with atopic dermatitis. J Dermatol Sci.

[29] Mouries J, Moron G, Schlecht G, Escriou N, Dadaglio G, Leclerc C (2008). Plasmacytoid dendritic cells efficiently cross-prime naive T cells in vivo after TLR activation. Blood.

[30] Young LJ, Wilson NS, Schnorrer P, Proietto A, ten Broeke T, Matsuki Y, Mount AM, Belz GT, O'Keeffe M, Ohmura-Hoshino M, Ishido S, Stoorvogel W, Heath WR, Shortman K, Villadangos JA (2008). Differential MHC class II synthesis and ubiquitination confers distinct antigen-presenting properties on conventional and plasmacytoid dendritic cells. Nat Immunol.

[31] Duez C, Gosset P, Tonnel AB (2006). Dendritic cells and toll-like receptors in allergy and asthma. Eur J Dermatol.

[32] Allam JP, Wurtzen PA, Reinartz M, Winter J, Vrtala S, Chen KW, Valenta R, Wenghoefer M, Appel T, Gros E, Niederhagen B, Bieber T, Lund K, Novak N (2010). Phl p 5 resorption in human oral mucosa leads to dose-dependent and time-dependent allergen binding by oral mucosal Langerhans cells, attenuates their maturation, and enhances their migratory and TGF-beta1 and IL-10-producing properties. J Allergy Clin Immunol.

[33] Charbonnier AS, Hammad H, Gosset P, Stewart GA, Alkan S, Tonnel AB, Pestel J (2003). Der p 1-pulsed myeloid and plasmacytoid dendritic cells from house dust mite-sensitized allergic patients dysregulate the T cell response. J Leukoc Biol.

[34] An HJ, Kim JY, Kim WH, Gwon MG, Gu HM, Jeon MJ, Han SM, Pak SC, Lee CK, Park IS, Park KK (2018). Therapeutic effects of bee venom and its major component, melittin, on atopic dermatitis in vivo and in vitro. Br J Pharmacol.

[35] Lei DK, Grammer LC (2019). An overview of allergens. Allergy Asthma Proc.

[36] Gattinger P, Bidovec-Stojkovic U, Zidarn M, Korosec P, Valenta R, Mittermann I (2021). Glycosylation enhances allergenic activity of major bee venom allergen Api m 1 by adding IgE epitopes. J Allergy Clin Immunol.

[37] Harvey DJ, Wing DR, Kuster B, Wilson IB (2000). Composition of N-linked carbohydrates from ovalbumin and co-purified glycoproteins. J Am Soc Mass Spectrom.

[38] Maldonado S, Fitzgerald-Bocarsly P (2017). Antifungal activity of plasmacytoid dendritic cells and the impact of chronic HIV infection. Front Immunol.

[39] Deslee G, Charbonnier AS, Hammad H, Angyalosi G, Tillie-Leblond I, Mantovani A, Tonnel AB, Pestel J (2002). Involvement of the mannose receptor in the uptake of Der p 1, a major mite allergen, by human dendritic cells. J Allergy Clin Immunol.

[40] Emara M, Royer PJ, Mahdavi J, Shakib F, Ghaemmaghami AM (2012). Retagging identifies dendritic cell-specific intercellular adhesion molecule-3 (ICAM3)-grabbing non-integrin (DC-SIGN) protein as a novel receptor for a major allergen from house dust mite. J Biol Chem.

[41] Mukhopadhyay A, Stahl P (1995). Bee venom phospholipase A2 is recognized by the macrophage mannose receptor. Arch Biochem Biophys.

[42] Sallusto F, Cella M, Danieli C, Lanzavecchia A (1995). Dendritic cells use macropinocytosis and the mannose receptor to concentrate macromolecules in the major histocompatibility complex class II compartment: downregulation by cytokines and bacterial products. J Exp Med.

[43] Royer PJ, Emara M, Yang C, Al-Ghouleh A, Tighe P, Jones N, Sewell HF, Shakib F, Martinez-Pomares L, Ghaemmaghami AM (2010). The mannose receptor mediates the uptake of diverse native allergens by dendritic cells and determines allergen-induced T cell polarization through modulation of IDO activity. J Immunol.

[44] Kaisho T, Akira S (2006). Toll-like receptor function and signaling. J Allergy Clin Immunol.

[45] Fabricius D, Trzaska T, Jahrsdörfer B (2016). Granzyme B produced by plasmacytoid dendritic cells promotes antigen uptake while suppressing premature T cell activation. Int J Vaccine Res.

[46] Aryan Z, Holgate ST, Radzioch D, Rezaei N (2014). A new era of targeting the ancient gatekeepers of the immune system: toll-like agonists in the treatment of allergic rhinitis and asthma. Int Arch Allergy Immunol.

[47] Johansen P, Senti G, Martinez Gomez JM, Storni T, von Beust BR, Wuthrich B, Bot A, Kundig TM (2005). Toll-like receptor ligands as adjuvants in allergen-specific immunotherapy. Clin Exp Allergy.

[48] Hessel EM, Chu M, Lizcano JO, Chang B, Herman N, Kell SA, Wills-Karp M, Coffman RL (2005). Immunostimulatory oligonucleotides block allergic airway inflammation by inhibiting Th2 cell activation and IgE-mediated cytokine induction. J Exp Med.

[49] Tel J, Sittig SP, Blom RA, Cruz LJ, Schreibelt G, Figdor CG, de Vries IJ (2013). Targeting uptake receptors on human plasmacytoid dendritic cells triggers antigen cross-presentation and robust type I IFN secretion. J Immunol.

[50] Tel J, Lambeck AJ, Cruz LJ, Tacken PJ, de Vries IJ, Figdor CG (2010). Human plasmacytoid dendritic cells phagocytose, process, and present exogenous particulate antigen. J Immunol.

[51] Benitez-Ribas D, Tacken P, Punt CJ, de Vries IJ, Figdor CG (2008). Activation of human plasmacytoid dendritic cells by TLR9 impairs Fc gammaRII-mediated uptake of immune complexes and presentation by MHC class II. J Immunol.

[52] Almunia C, Bretaudeau M, Held G, Babon A, Marchetti C, Castelli FA, Menez A, Maillere B, Gillet D (2013). Bee venom phospholipase A2, a good "chauffeur" for delivering tumor antigen to the MHC I and MHC II peptide-loading compartments of the dendritic cells: the case of NY-ESO-1. PLoS ONE.

[53] Babon A, Wurceldorf T, Almunia C, Pichard S, Chenal A, Buhot C, Beaumelle B, Gillet D (2016). Bee venom phospholipase A2 as a membrane-binding vector for cell surface display or internalization of soluble proteins. Toxicon.

[54] Hartmann G, Weiner GJ, Krieg AM (1999). CpG DNA: a potent signal for growth, activation, and maturation of human dendritic cells. Proc Natl Acad Sci U S A.

[55] Cella M, Facchetti F, Lanzavecchia A, Colonna M (2000). Plasmacytoid dendritic cells activated by influenza virus and CD40L drive a potent TH1 polarization. Nat Immunol.

[56] Jacquet A (2013). Innate immune responses in house dust mite allergy. ISRN Allergy.

[57] Tel J, Torensma R, Figdor CG, de Vries IJ (2011). IL-4 and IL-13 alter plasmacytoid dendritic cell responsiveness to CpG DNA and herpes simplex virus-1. J Invest Dermatol.

[58] Bratke K, Klein C, Kuepper M, Lommatzsch M, Virchow JC (2011). Differential development of plasmacytoid dendritic cells in Th1- and Th2-like cytokine milieus. Allergy.

[59] Golden DB, Demain J, Freeman T, Graft D, Tankersley M, Tracy J, Blessing-Moore J, Bernstein D, Dinakar C, Greenhawt M, Khan D, Lang D, Nicklas R, Oppenheimer J, Portnoy J, Randolph C, Schuller D, Wallace D (2017). Stinging insect hypersensitivity: a practice parameter update 2016. Ann Allergy Asthma Immunol.

[60] Shaker MS, Wallace DV, Golden DBK, Oppenheimer J, Bernstein JA, Campbell RL, Dinakar C, Ellis A, Greenhawt M, Khan DA, Lang DM, Lang ES, Lieberman JA, Portnoy J, Rank MA, Stukus DR, Wang J, Collaborators, Riblet N, Bobrownicki AMP, Bontrager T, Dusin J, Foley J, Frederick B, Fregene E, Hellerstedt S, Hassan F, Hess K, Horner C, Huntington K, Kasireddy P, Keeler D, Kim B, Lieberman P, Lindhorst E, McEnany F, Milbank J, Murphy H, Pando O, Patel AK, Ratliff N, Rhodes R, Robertson K, Scott H, Snell A, Sullivan R, Trivedi V, Wickham A, Chief E, Shaker MS, Wallace DV, Workgroup C, Shaker MS, Wallace DV, Bernstein JA, Campbell RL, Dinakar C, Ellis A, Golden DBK, Greenhawt M, Lieberman JA, Rank MA, Stukus DR, Wang J, Joint Task Force on Practice Parameters R, Shaker MS, Wallace DV, Golden DBK, Bernstein JA, Dinakar C, Ellis A, Greenhawt M, Horner C, Khan DA, Lieberman JA, Oppenheimer J, Rank MA, Shaker MS, Stukus DR, Wang J: Anaphylaxis-a 2020 practice parameter update, systematic review, and Grading of Recommendations, Assessment, Development and Evaluation (GRADE) analysis. *J Allergy Clin Immunol* 2020, 145:1082-1123 10.1016/j.jaci.2020.01.017 32001253

[61] Bessot JC, Pauli G (2011). Mite allergens: an overview. Eur Ann Allergy Clin Immunol.

[62] Kitzmuller C, Wallner M, Deifl S, Mutschlechner S, Walterskirchen C, Zlabinger GJ, Ferreira F, Bohle B (2012). A hypoallergenic variant of the major birch pollen allergen shows distinct characteristics in antigen processing and T-cell activation. Allergy.

[63] Dreschler K, Bratke K, Petermann S, Bier A, Thamm P, Kuepper M, Virchow JC, Lommatzsch M (2011). Impact of immunotherapy on blood dendritic cells in patients with Hymenoptera venom allergy. J Allergy Clin Immunol.

[64] Sousa L, Martin-Sierra C, Pereira C, Loureiro G, Tavares B, Pedreiro S, Martinho A, Paiva A (2018). Subcutaneous immunotherapy induces alterations in monocytes and dendritic cells homeostasis in allergic rhinitis patients. Allergy Asthma Clin Immunol.

[65] Uto T, Takagi H, Fukaya T, Nasu J, Fukui T, Miyanaga N, Arimura K, Nakamura T, Choijookhuu N, Hishikawa Y, Sato K (2018). Critical role of plasmacytoid dendritic cells in induction of oral tolerance. J Allergy Clin Immunol.

