# Supplementary material for: Differential uptake of three clinically relevant allergens by human plasmacytoid dendritic cells
Source: Clin Mol Allergy. 2021 Nov 17;19:23. doi: 10.1186/s12948-021-00163-8 (PMC8597288; doi:10.1186/s12948-021-00163-8)
Supplement: Supplementary file 1 — Additional file 1: Figure S1. Gating strategy of pDC analysis. PDC isolated from fresh peripheral blood, buffy coats or leukapheresis products by BDCA-4 positive magnetic bead selection were analyzed at day 0 for purity using flow cytometry. For this purpose, cells were stained with Lin-1 FITC, a lineage cocktail including antibodies to CD3, CD14, CD19, CD20, CD56) and with anti-BDCA-2 PE, with pDC being defined as Lin-1neg/BDCA-2pos cells. If purity was > 95% pDC were analyzed without further gating after culture. If purity was <95%, cultured pDC, which tend to downregulate BDCA-2, were stained with Lin-1 FITC and anti-CD123 PerCPCy5.5 and gated as Lin-1neg/ CD123highly pos cells. Figure S2. CpG-ODN and IL-4 suppress OVA-uptake. PDC isolated from leukapheresis products by BDCA-4 positive magnetic bead selection with a purity of 95% were cultured in 10 ng/ml IL-3-containing AIM-V medium for 16h in the presence or absence of allergens, CpG ODN 2006 (2.5 μg/ml), IL-4 (500 U/ml) or IL-13 (100 U/ml). Allergens included DQ OVA Bodipy or Alexa Fluor 647-labeled OVA at a final concentration of 30 μg/ml. After incubation, OVA uptake (OVA Alexa 647) and OVA processing (DQ OVA Bodipy) was quantified using flow cytometry. (A) Dot plots show one out of at least 6 representative experiments. (B) Box plots show percentage of allergen-positive pDC, central horizontal lines indicate medians, box borders represent IQR, whiskers indicate minima and maxima, n = 5-12 experiments. Significance levels were **** p < 0.00005 and * p < 0.05, assessed with Kruskal-Wallis test, followed by Dunn‘s multiple comparisons test. Figure S3. Allergen exposure has only minor effects on pDC phenotype. Isolated pDC were incubated for 24 h in 10 ng/ml IL-3 containing AIM-V medium with Der p 1 (20 μg/ml) or Api (bee venom extract, 10 μg/ml), IL-4 (500 U/ml) or IL-13 (100 U/ml) or combinations as indicated and measured by flow cytometry. CD40, CD80, CD83, CD86 , MHC class I and MHC class II expression is s [file 12948_2021_163_MOESM1_ESM.pdf]

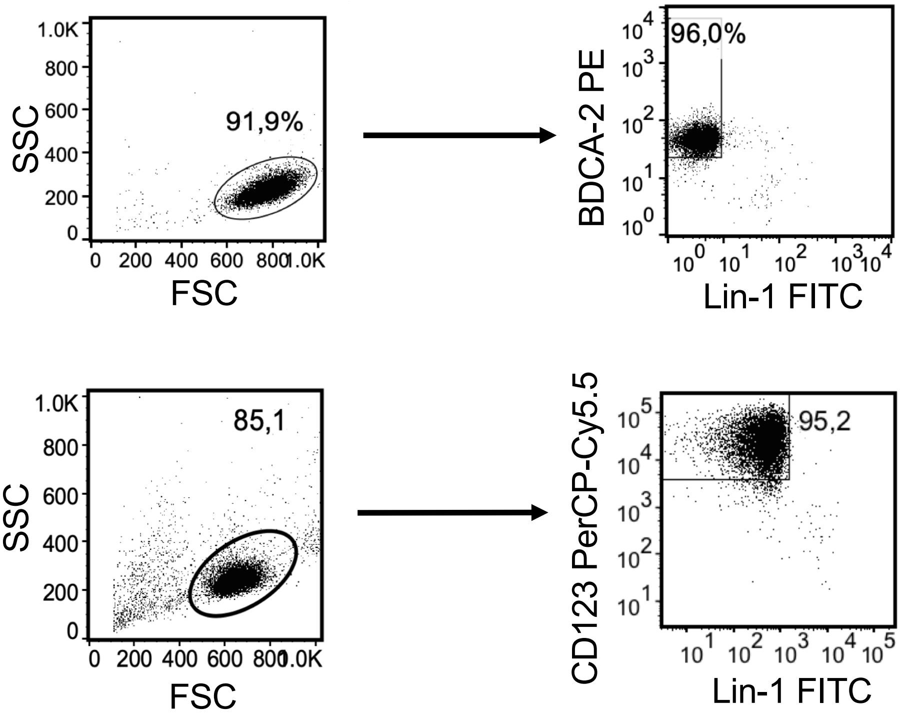

Supplemental figure 1. Gating strategy of pDC analysis. PDC isolated from fresh peripheral blood, buffy coats or leukapheresis products by BDCA-4 positive magnetic bead selection were analyzed at day 0 for purity using flow cytometry. For this purpose, cells were stained with Lin-1 FITC, a lineage cocktail including antibodies to CD3, CD14, CD19, CD20, CD56) and with anti-BDCA-2 PE, with pDC being defined as Lin-1^{neg}/BDCA-2^{pos} cells. If purity was > 95% pDC were analyzed without further gating after culture. If purity was <95%, cultured pDC, which tend to downregulate BDCA-2, were stained with Lin-1 FITC and anti-CD123 PerCPCy5.5 and gated as Lin-1^{neg}/ CD123$^{highly\ pos}$ cells.

A

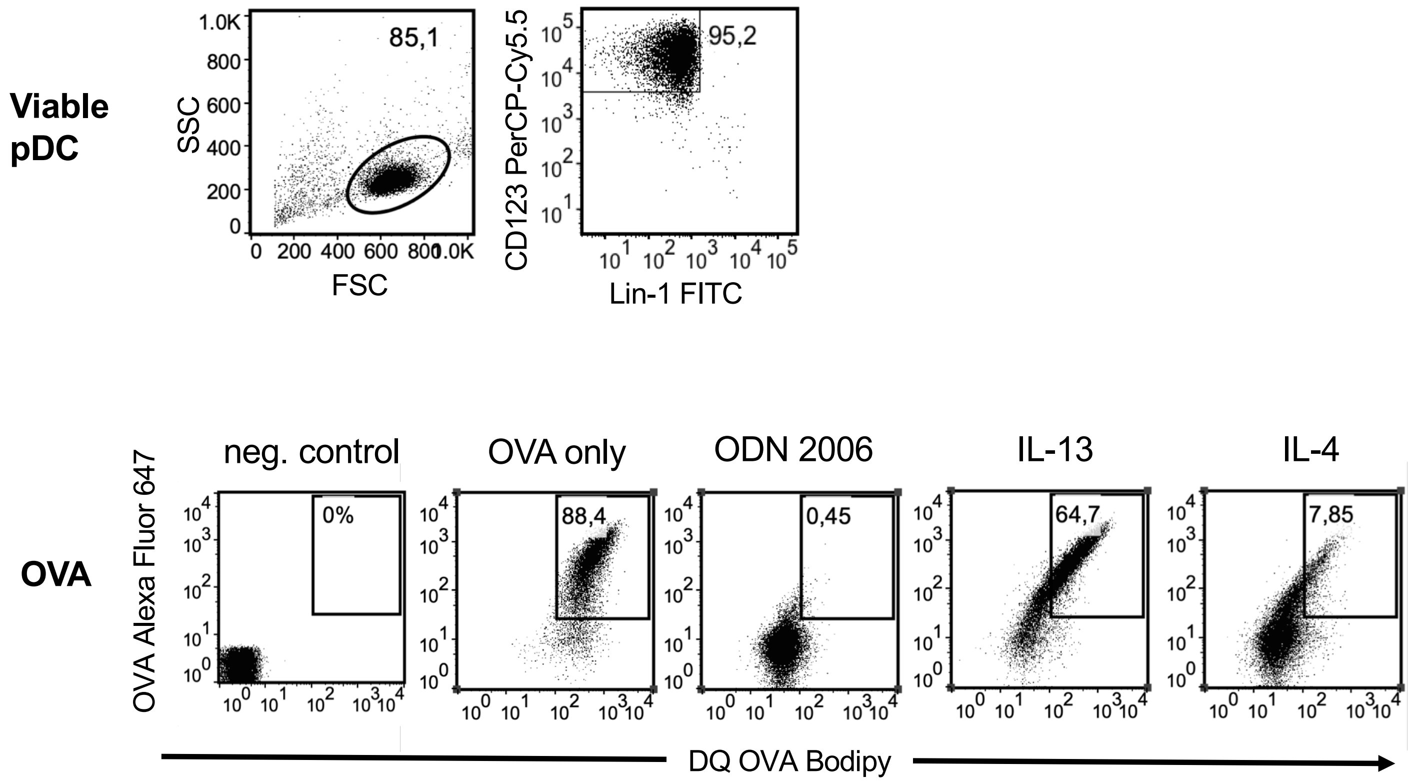

B

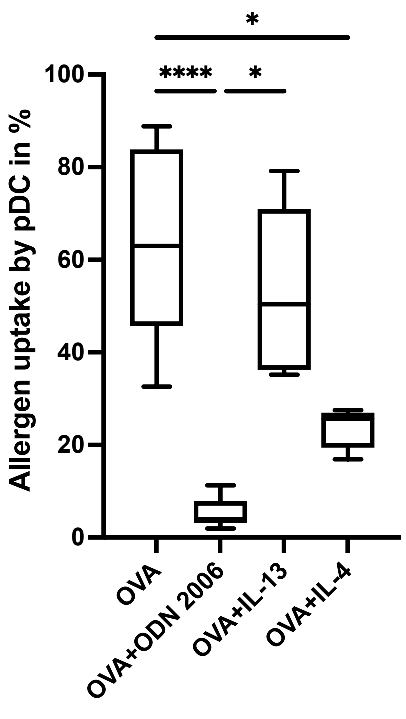

Supplemental figure 2. CpG-ODN and IL-4 suppress OVA-uptake. PDC isolated from leukapheresis products by BDCA-4 positive magnetic bead selection with a purity of 95% were cultured in 10 ng/ml IL-3-containing AIM-V medium for 16h in the presence or absence of allergens, CpG ODN 2006 (2.5 µg/ml), IL-4 (500 U/ml) or IL-13 (100 U/ml). Allergens included DQ OVA Bodipy or Alexa Fluor 647-labeled OVA at a final concentration of 30 µg/ml. After incubation, OVA uptake (OVA Alexa 647) and OVA processing (DQ OVA Bodipy) was quantified using flow cytometry. (**A**) Dot plots show one out of at least 6 representative experiments. (**B**) Box plots show percentage of allergen-positive pDC, central horizontal lines indicate medians, box borders represent IQR, whiskers indicate minima and maxima, n = 5-12 experiments. Significance levels were **** $p < 0.00005$ and * $p < 0.05$, assessed with Kruskal-Wallis test, followed by Dunn‘s multiple comparisons test.

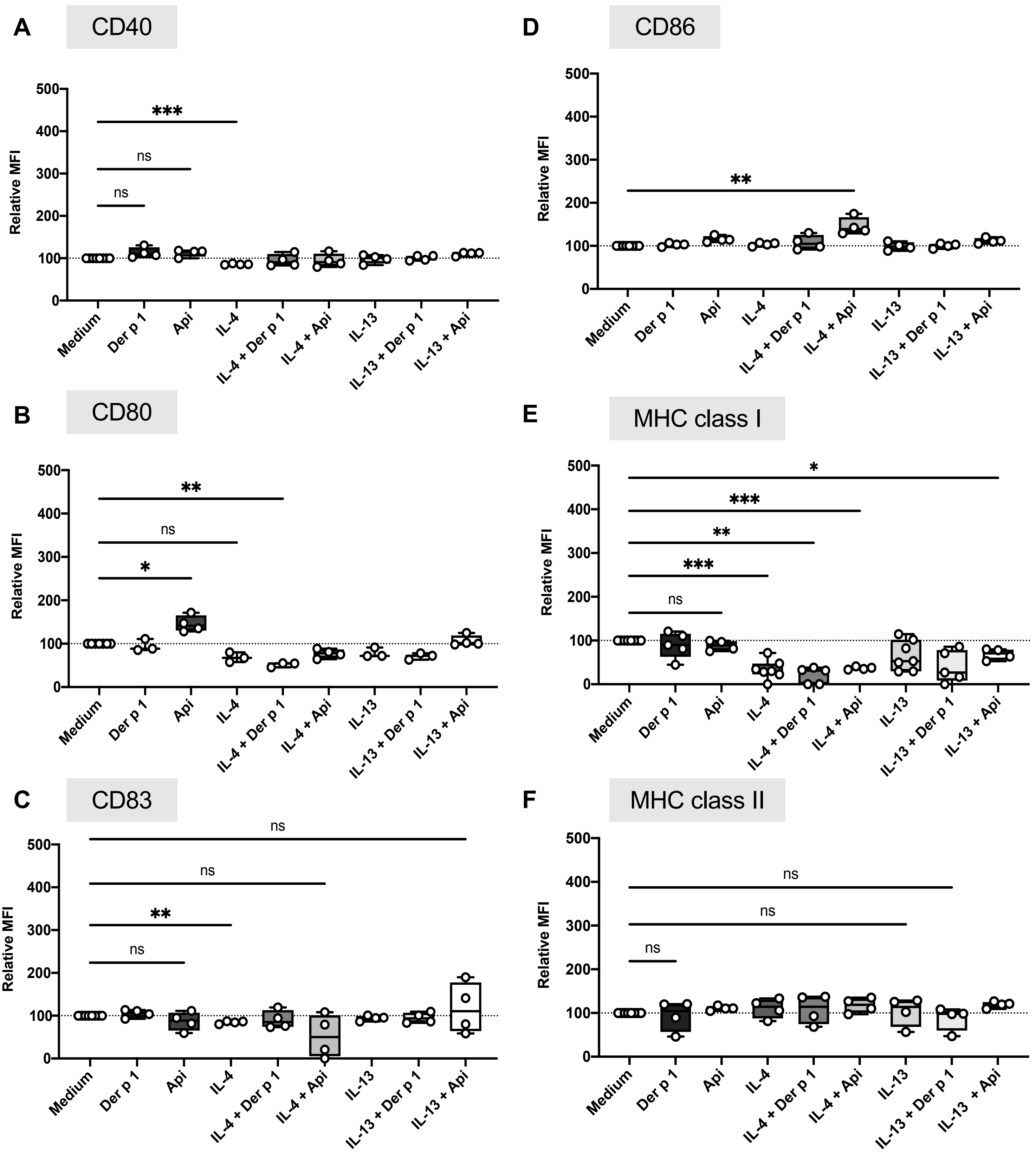

Supplemental figure 3. Allergen exposure has only minor effects on pDC phenotype. Isolated pDC were incubated for 24 h in 10 ng/ml IL-3 containing AIM-V medium with Der p 1 (20 µg/ml) or Api (bee venom extract, 10 µg/ml), IL-4 (500 U/ml) or IL-13 (100 U/ml) or combinations as indicated and measured by flow cytometry. CD40, CD80, CD83, CD86 , MHC class I and MHC class II expression is shown as mean fluorescence intensity (MFI) relative to non-stimulated cells (medium), box plot central lines represent medians, box borders show IQR, whiskers indicate minima and maxima, dots represent individual values of at least 3 independent experiments. Significant differences between medium and various stimuli were assessed with Kruskal-Wallis test or Mixed effects analysis, followed by Dunett's multiple comparisons test comparing all culture conditions to non-stimulated cells (medium), significance level was $p < 0.05$.
